# Polystyrene Microsphere-Based Immunochromatographic Assay for Detection of Aflatoxin B_1_ in Maize

**DOI:** 10.3390/bios11060200

**Published:** 2021-06-20

**Authors:** Jin Wang, Xiangmei Li, Xing Shen, Ang Zhang, Jinxiu Liu, Hongtao Lei

**Affiliations:** 1 Guangdong Provincial Key Laboratory of Food Quality and Safety, South China Agricultural University, Guangzhou 510642, China; wangjin940810@stu.scau.edu.cn (J.W.); lixiangmei12@163.com (X.L.); shenxing325@163.com (X.S.); ljx199108z@163.com (J.L.); 2 Technology Center of Qinhuangdao Customs, Qinhuangdao 066004, China; zhanganggrape@hotmail.com; 3 Guangdong Laboratory for Lingnan Modern Agriculture, Guangzhou 510642, China

**Keywords:** polystyrene microspheres, immunochromatographic assay, aflatoxin B_1_, maize

## Abstract

Aflatoxin B_1_ (AFB_1_), a mycotoxin, is hepatotoxic, carcinogenic, and nephrotoxic in humans and animals, and contaminate a wide range of maize. In this study, an immunochromatographic assay (ICA) based on polystyrene microspheres (PMs) was developed for sensitive and quantitative detection of AFB_1_ in maize. The amounts of PMs, the condition for activating carboxyl groups of PMs, the amount of monoclonal antibody (mAb), and the volume of the immune probe were optimized to enhance the performance PMs-ICA for point-of-care testing of AFB_1_ in maize. The PMs-ICA showed the cut-off value of 1 ng/mL in phosphate buffer (PB) and 6 µg/kg in maize samples, respectively. The quantitative limit of detection (qLOD) was 0.27 and 1.43 µg/kg in PB and maize samples, respectively. The accuracy and precision of the PMs-ICA were evaluated by analysis of spiked maize samples with recoveries of 96.0% to 107.6% with coefficients of variation below 10%. In addition, the reliability of PMs-ICA was confirmed by the liquid chromatography-tandem mass spectrometry method. The results indicated that the PMs-ICA could be used as a sensitive, simple, rapid point-of-care testing of AFB_1_ in maize.

## 1. Introduction

Aflatoxins (AFTs), a class of mycotoxins, are toxic and carcinogenic secondary metabolites produced by *A. flavus, A. parasiticus,* and the rare *A. nomius* [1]. Aflatoxin B_1_ (AFB_1_), aflatoxin B_2_ (AFB_2_), aflatoxin G_1_ (AFG_1_), and aflatoxin G_2_ (AFG_2_) are the common AFTs [2], and AFB_1_ presents hepatotoxic, carcinogenic, and nephrotoxic in humans and animals [3,4,5]. The International Agency for Research on Cancer (IAMC) listed AFB_1_ as a Group I carcinogen [6]. AFB_1_ demonstrated high chemical stability against elevated temperature through food processing, making the prevention of their entrance into the food supply chain difficult [7]. To ensure food safety and protect human health, the maximum allowable limits of AFB_1_ in maize have been set by many countries and regions. For examples, 20 µg/kg is the maximum allowable limits in maize and its products in China (GB 2761-2017) and 2 µg/kg is the maximum limit for peanuts and cereals in the European Commission (EC) [8]. However, a recent survey showed that AFB_1_ had a high prevalence in cereals, which, in most of the cases, it exceeded the EC allowed limit [9]. In order to ensure the concentration of AFB_1_ in maize is less than the maximum allowable limits, it is necessary to develop an accurate, effective detection method to monitor the concentration of AFB_1_. Currently, the common methods for AFB_1_ detection include instrumental analysis methods and immunoassay. Instrumental analysis methods, such as Fourier transform infrared spectroscopy (FTIR) [10], high-performance liquid chromatography (HPLC) [11,12,13], liquid chromatography-tandem mass spectrometry (LC–MS/MS) [14,15], etc., provide accurate and reliable quantitative detection results. However, they need expensive equipment, complex sample preparation, professional skilled personnel, and much time. Moreover, these techniques are not suitable for point-of-caring detection. For the immunoassay, enzyme-linked immunosorbent assay (ELISA) and lateral flow immunochromatographic assay (LFICA) are common methods. LFICA, as a typical point-of-care technology [16], has been widely applied to food analysis [17,18], environmental monitoring [19], and in vitro diagnosis [20,21]. Comparing the ELISA, LFICA is used for the detection of AFB_1_, which is rapid, low cost, simple detection steps, and more suitable for a large number of samples screening [22,23,24]. Colloidal gold and fluorescent microspheres (FMs) are the two most common label materials for LFICA [25,26]. However, CG-LFICA is often limited by sensitivity [27], and FMs-LFICA has the advantages in sensitivity and signal intensity, but it requires a fluorescence reader to obtain results [28]. In order to obtain high sensitivity and brighter colors than CG-ICA, the detection results can be obtained by the naked eye instead of a reader similar to FMs-ICA. In the study, based on PMs, an ICA was developed for sensitive and quantitative detection of AFB_1_ in maize. The principle of PMs-ICA is based on the completive reaction among the coating antigen (AFB_1_-BSA) coated on the T line, the analyte in the samples and the polystyrene microspheres-monoclonal antibody (PMs-mAb) conjugate. The amounts of PMs, the condition for activating carboxyl groups of PMs, the amount of mAb, and the volume of the immune probe were optimized to enhance the performance PMs-ICA for point-of-care testing of AFB_1_ in maize. The developed detection method shows high sensitivity, good accuracy, and precision for detection of AFB_1_ in maize.

## 2. Materials and Methods

### 2.1. Reagents and Instruments

Aflatoxin B_1_, 2-(N-morpholino) ethanesulfonic acid, 1-ethyl-3-(3-dimethylaminopropyl)-carbodiimide (EDC), *N*-hydroxysuccinimide (NHS), bovine serum albumin (BSA), 4-morpholineethanesulfonic acid (MES) were purchased from Sigma-Aldrich (St. Louis, MO, USA). Dye polystyrene microspheres (0.2000 µm, crimson red, 50 mg/mL) were obtained from Bangs Laboratories, Inc. (Fishers, IN, USA). The nitrocellulose filter (NC) membrane (Sartorius, UniSart CN95, Goettingen, Germany) was purchased form Sartorius Stedim Biotech GmbH (Göttingen, Germany). The polyvinylchloride (PVC) backing plate (SMA31-40), sample pad (SB08), and absorbent pad were obtained from Shanghai Kinbio Tech. Co., Ltd. (Shanghai, China). Other chemical substances were purchased from the Damao Chemical Reagent Factory (Tianjin, China). The XYZ^TM^ Dispense Platform comprised motion control with Biostrip Dispenser HGS102 and Airjet HGS102 was purchased from BioDot Inc. (Irvine, CA, USA). Programmable Sheet Cutter and Programmable strip cutter were purchased from Shanghai Kinbio Tech. Co., Ltd. (Shanghai, China). The GL-23M Centrifuge was provided by Xiangyi Centrifuge Instrument Co., Ltd. (Changsha, China), HP ScanJet g3110 was supplied by HP China. (Shanghai, China). Anti-AFB_1_ mAb and coating antigen (AFB_1_-BSA) were prepared in our laboratory (Preparation of coating antigen and Anti-AFB1 mAb were described in Supplementary File).

### 2.2. Labeling of mAbs with PMs

**Figure 1:**
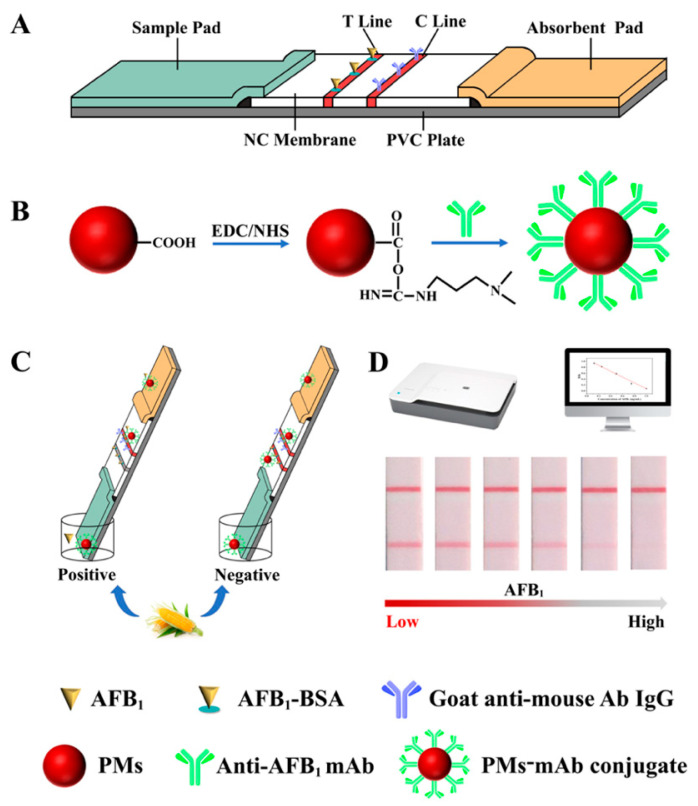
The scheme of the PMs-ICA. (**A**) The structure of the test strips, including a sample pad, a NC membrane, an absorbent pad, a PVC plate, and the AFB_1_-BSA, and goat anti-mouse antibody IgG were coated on the NC membrane form the T line and C line, respectively. (**B**) The process of labeling of mAbs with PMs. (**C**) Qualitative detection of the PMs-ICA. (**D**) Quantitative detection of the PMs-ICA.

The EDC activated ester method was used to couple carboxy-modified PMs with the mAbs [29]. The principle of conjugation is presented in Figure 1B. Initially, PMs were suspended in 1 mL of MES or PBS solution, then 15 µL of EDC (0.05 mg/mL) and 18 µL of NHS (0.05 mg/mL) solution were added to the above solution, and stirred with a shaker at 200 rpm for 15 min to active carboxyl groups of PMs. After activation, the mixture was centrifuged at 14,000 rpm for 15 min at 4 °C to discard the excess activation solution and the precipitate was dissolved in borate buffer [30] solution (0.05 mol/L, pH 8.0) solution. The mAb was added and coupled with PMs and shaken at 200 rpm for 30 min; 40 µL of BSA (10%, *w/v*) was then added to block the sites on the surface of PMs under shaking. After conjugation, the mixture was centrifuged at 14,000 rpm for 15 min at 4 °C, and repeated twice to discard the supernatant. The PMs-mAb conjugate was dissolved in PBS (0.01 mol/L, pH 7.4) containing BSA (0.5%, *w/v*) and Tween-20 (0.05%, *v/v*), and was stored at 4 °C for future use.

### 2.3. Fabrication of PMs-ICA Strips

The PMs-ICA strip consists of a sample pad, a NC membrane, an absorbent pad, and a PVC plate (Figure 1A). The appropriate concentration of coating antigen (0.25 mg/mL, AFB_1_-BSA) and goat anti-mouse IgG antibody (0.4 mg/mL), diluted by the coating buffer (PBS, 0.01 mol/L, pH 7.4), were coated onto the NC membrane to form the test line (T line) and control line (C line), with the dispense platform (XYZ 3060, BioDot, Irvine, CA, USA) at a jetting rate of 0.8 µL/cm. Then, the NC membrane was dried at 37 °C overnight. The sample pad was pretreated with 0.5% sucrose, 0.3% polyvinylpyrrolidone, 0.5% BSA, and 0.5% Tween-20 in PB solution (0.05 mol/L, pH 7.4), and dried at 37 °C overnight. The sample pad and absorbent pad were cut into 15 and 25 cm, respectively. Subsequently, the NC membrane, the sample pad, and the absorbent pad were sequentially pasted on the PVC plate with an overlap of 1–2 mm. Finally, the assembled plate was cut into 3.05 mm wide strips and kept in a desiccator at room temperature.

### 2.4. Optimization of Key Parameters

To evaluate the influence of the amounts of PMs on the performance of PMs-ICA and obtain appreciate amounts of PMs to couple with anti-AFB_1_ mAb, different amounts of PMs were compared, including 5, 10, 15, 20, 25, 30 µL. The negative control groups (PBS, 0.01 mol/L, pH 7.4) and positive test groups (1 ng/mL AFB_1_) were tested at the same condition.

To activate carboxyl groups on the surface of PMs more efficiently, six buffer solution were selected to be tested, including 0.05 mol/L MES (pH 5, 5.5, 6, 6.5) and 0.01 mol/L PBS (pH 7.0, 7.4). The negative control groups (PBS, 0.01 mol/L, pH 7.4) and positive test groups (1 ng/mL AFB_1_) were tested at the same condition to evaluate the influence of the condition for activating carboxyl groups on the surface of PMs.

The amount of mAb was optimized because it directly affected the color intensity of the T line, and the sensitivity of PMs-ICA. 2, 2.5, 3, 3.5, 4 µL of mAb (1 mg/mL) were selected to couple with PMs. The results of the color intensity of the T Line on the negative control groups (PBS, 0.01 mol/L, pH 7.4) and the inhibition effect on the positive test groups (1 ng/mL AFB_1_) were tested and considered simultaneously to determine the amount of optimal mAb.

Applying PMs-ICA to real samples, the volume of PMs-Ab conjugate (immune probe) was a key factor affecting the color intensity of the T line and the sensitivity of PMs-ICA. Moreover, 3, 4, 5, 6 µL of immune probes were added to tested and compared in spiked maize samples. The negative control groups (negative maize samples) and positive test groups (negative maize samples spiked 6 µg/kg AFB_1_) were tested to decide the volume of the immune probe.

### 2.5. Sample Preparation

A total of 5 g of maize samples to a 50 mL centrifuge tube, then 10 mL of methanol/water (7:3, *v/v*) was added, and the mixture was shaken for 2 min on a vortex shaker. After the mixture was centrifuged at 5000 rpm for 5 min, and the supernatant was collected and filtered through 0.22 µm filter, 100 µL of supernatant was added to 200 µL of PB (0.02 mol/L, pH 7.4) for detection.

### 2.6. Test Procedure

A total of 150 µL of standard or sample solution and a certain amount of PMs-mAbs conjugates were mixed into microplate wells and incubated for 3 min at room temperature. Then, the prepared test strips were vertically inserted into the micropores for another 5 min chromatographic reaction, and the sample pad was rejected immediately. For qualitative detection, the results of ICA strips can be observed by the naked eye. For quantitative detected, the ICA strips were scanned by an HP ScanJet (HP ScanJet g3110, Shanghai, China), and the digital images of strips were analyzed by the TotalLab TL120 software (Nonlinear Dynamics, Newsletter, UK) [31].

### 2.7. Sensitivity and Specificity of PMs-ICA

A series of concentrations of AFB_1_, including 0, 0.125, 0.25, 0.5. 0.75, 1 ng/mL were prepared, the sensitivity of PMs-ICA was evaluated under optimal condition, and each concentration of AFB_1_ was detected by three parallel experiments. For qualitative detection, the cut-off value was defined as the concentration of AFB_1_, resulting in no red bands on the T line judged by the naked eye [32]. For quantitative detection, the digital images scanned with a scanner were analyzed by TotalLab TL120 software. B/B0 was calculated and the calibration curve fitted by OriginPro 9.1 software (OriginLab Corp., Northampton, MA, USA) was obtained by plotting B/B0 against the concentration of AFB_1_. B and B0 denoted the color intensity of the T line in the positive control group and the test negative group, respectively. The qLOD was defined as the concentration giving 80% B/B_0_ from the calibration curve [33,34]. For the sensitivity of PMs-ICA in maize samples, the negative maize samples were brought from the local market and spiked. The sensitivity was evaluated under optimal conditions, with AFB_1_ at concentrations of 0, 0.75, 1.5, 3, 4.5, 6 µg/kg.

To evaluate specificity, expressed as cross-reactivity (CR, %), of PMs-ICA for AFB_1_ detection, other mycotoxins found in maize, such as Aflatoxin B_2_ (AFB_2_), Aflatoxin G_1_ (AFG_1_), Aflatoxin G_2_ (AFG_2_), Aflatoxin M_1_ (AFM_1_) zearalenone (ZEN), ochratoxin A (OTA), deoxynivalenol (DON), fumonisin B_1_ (FB_1_), T-2, were tested in PB.CR (%), expressed as the percentage of the IC_50_ value of the target analyte to analogues [33].

### 2.8. Accuracy and Precision

The accuracy (expressed as recovery) and precision (expressed as coefficient of variation (CV)) of the PMs-ICA were evaluated, and were determined after spiking with AFB_1_ at concentrations of 1, 3, 5, 12 µg/kg in maize samples; the results were detected after sample preparation.

### 2.9. Detection of Real Maize Samples

To evaluate the reliability of PMs-ICA, 20 real maize samples brought from the local market and provided by Shanxi Institute of Feed and Veterinary Drug control were detected by PMs-ICA and LC–MS/MS. The LC–MS/MS method was performed according to our lab’s previous study, with slight modifications [35]. Briefly, the dry grated samples (10.0 g) were extracted with 40.0 mL of acetonitrile/water/acetic acid (79:20:1, *v/v*) by vigorous stirring for 10 min. Then, the mixture was centrifuged at 5000 g for 10 min. Purification of the extract was conducted using 0.5 mL of the final extract diluted with same amount of acetonitrile/water/acetic acid (20:79:1, *v/v*), and then a second purification was conducted using a syringe filter (0.22 μm). Chromatographic separation was conducted with an Agilent C_18_ column (InfinityLab Poroshell 120 EC-C18, 4.6 × 150 mm 2.7-Micron. The mobile phase consisted of 0.1% acetic acid (mobile phase A) and 100% methanol (mobile phase B), and the mobile phase flow rate was 700 μL/min. The gradient elution procedure was carried as follows: 0–5 min, 5–90% B; 5–7 min, 90% B; 7–7.1 min, 90–5% B, 7.1–10 min, 5% B. The column was kept at 30 and the sample injection volume was 10 μL. Mass analyses were carried out by electrospray ionization (ESI) sources in positive-ion mode. The spray voltage was 5.5 kV. The capillary temperature was set at 550 °C. Curtain gas, spray gas, and auxiliary gas maintained at a pressure of 30, 50 and 50 psi, respectively.

## 3. Results and Discussion

### 3.1. Principle of PMs-ICA

Figure 1 illustrates the schematic process of detection AFB_1_ by PMs-ICA. Figure 1A presents the structure of the test strips, including a sample pad, a NC membrane, an absorbent pad, and a PVC plate; the AFB_1_-BSA and goat anti-mouse antibody IgG were coated on the NC membrane form the T line and C line, respectively. Figure 1A shows the process of labeling of mAbs with PM, the carboxyl groups of PMs were firstly activated by EDC solution, then the mAb was added to couple with PMs. Based on the competitive inhibition interaction between the free AFB_1_ in the samples and the coating antigen (AFB_1_-BSA) spaced on the NC membrane, the PMs-ICA was developed. If there was no AFB_1_ in the samples, the PMs-Ab conjugates bound to the antigen coated on the T Line to form a red band (Figure 1C,D). If there was AFB_1_ in the samples, AFB_1_ bound to the PMs-Ab conjugates to form a PMs-Ab-AFB_1_ complex in the well, and the binding sites of mAb would be firstly occupied by AFB_1_. The excessive PMs-Ab conjugates would be captured by the antigen, and the color intensity of T line would gradually decrease to zero with the concentration of AFB_1_ increase (Figure 1C,D). Regardless of whether there is AFB_1_ in the sample, PMs-mAb conjugate would pass through the T line and react with the goat anti-mouse antibody IgG coated on the C line, which was used to ensure the validity of the PMs-ICA.

### 3.2. Optimization of Key Parameters of PMs-ICA

#### 3.2.1. The Amount of PMs

**Figure 2:**
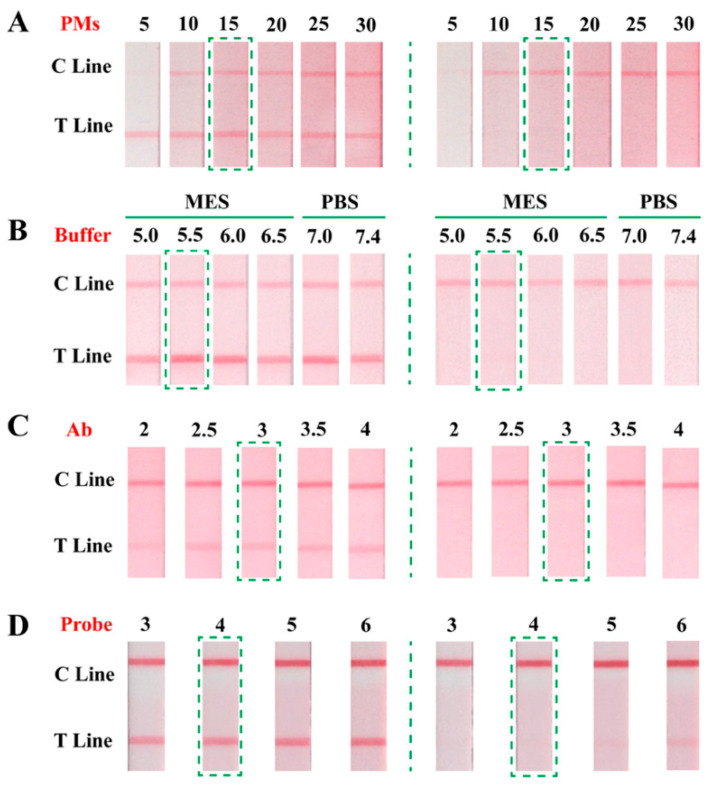
Optimization results of key parameters of PMs-ICA. (**A**) The amounts of PMs (5, 10, 15, 20, 25, 30). (**B**) The condition for activating carboxyl groups (0.05 mol/L MES pH 5, 5.5 6, 6.5; 0.01 mol/L PBS pH 7.0, 7.4. (**C**) The amount of mAb (2, 2.5, 3, 3.5, 4 µL). (**D**) The volume of immune probe (3, 4, 5, 6).

PMs, the label of the PMs-ICA, were directly related the color intensity of the T line. Hence, the amounts of PMs were optimized to get enough color intensity at the lowest cost. As shown in Figure 2A, for the negative control groups, the color intensity of the T line gradually enhanced until the amounts of PMs reached 15 µL, the color intensity of the T line was no longer enhanced. For positive test groups, the T line of each group was colorless. Considering the price of PMs, 15 µL of PMs were selected for the next optimization experiments.

#### 3.2.2. The Condition for Activating Carboxyl Groups of PMs

In order to enable PMs to couple with Ab, the carboxyl groups of PMs must be activated by the EDC method. The condition for activating carboxyl groups of PMs is an important factor [36], 0.05 mol/L MES (pH 5.0, 5.5, 6.0, 6.5) and 0.01 mol/L PBS (pH 7.0, 7.4) were prepared for the solution to active carboxyl groups. As presented in Figure 2B, no matter what the negative control groups or positive test groups were, the PMs-ICA had the best color intensity, and the same inhibition effect, activating by 0.5 mol/L MES (pH 5.5). Therefore, the MES (0.05 mol/L, pH 5.5) solution was chosen to activate carboxyl groups for next optimization experiments.

#### 3.2.3. The Amounts of mAb

The amounts of mAb directly affect the sensitivity of the immunoassay [32]; 2, 2.5, 3, 3.5, 4 µL of mAb were selected to couple with PMs, respectively, and the sensitivity was tested. For the negative control groups, the color intensity of the T line gradually increased as the amount of mAb increased, but the color intensity was (basically) stable when the amount of antibody was added, 3 µL, which should be due to the limited carboxyl groups on the surface of the PMs (Figure 2C). For the positive test groups, all T lines were colorless. To obtain the best result with the least amount of mAb, 3 µL of mAb was decide for the next optimization experiments.

#### 3.2.4. The Immune Probe Amount

Applying PMs-ICA to actual samples—the PMs-Ab conjugate was used as the immune probe and its volume would directly affect the performance of PMs-ICA. Hence, under the other optimal conditions, 3, 4, 5, 6 µL of the immune probe was added to the well for testing and comparing in spiked maize samples. The results of optimization are shown in Figure 2D, for the negative control groups, the color intensity of the T line gradually increased with the volume of the immune probe increasing. For the positive test groups, the T line appeared as a faint red band when the volume of the immune probe reached 4 µL. Considering the color intensity of the T line and the sensitivity of PMs-ICA, when the volume of the immune probe was 3 µL, the color intensity of the T line was enough and the sensitivity was better than the more immune probe. Therefore, 3 µL of the immune probe was added to the detected AFB_1_ in maize.

Furthermore, to reduce the time of labeling of mAb with PMs, the time of PMs couple with mAb, and the time of blocking PMs to bind the site to the mAb, were investigated (Figure S1). The length of time, the color intensity of the T line, the sensitivity, and the non-specific adsorption were comprehensively considered. The final optimal conditions of labeling of mAbs with PMs are summarized in Table S1.

### 3.3. Sensitivity and Specificity of PMs-ICA

**Figure 3:**
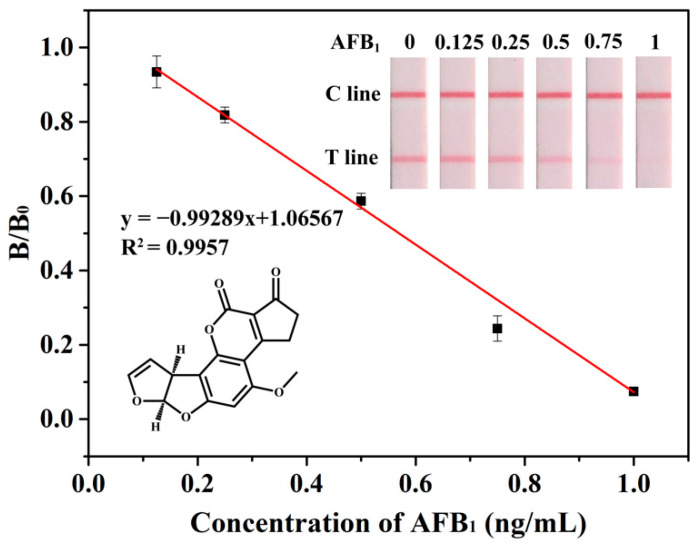
The sensitivity of PMs-ICA for detection of AFB_1_ in PB. Result for AFB_1_ standard of different concentrations, by the naked eye, and calibration curve of B/B_0_ against the concentration of AFB_1_.

**Figure 4:**
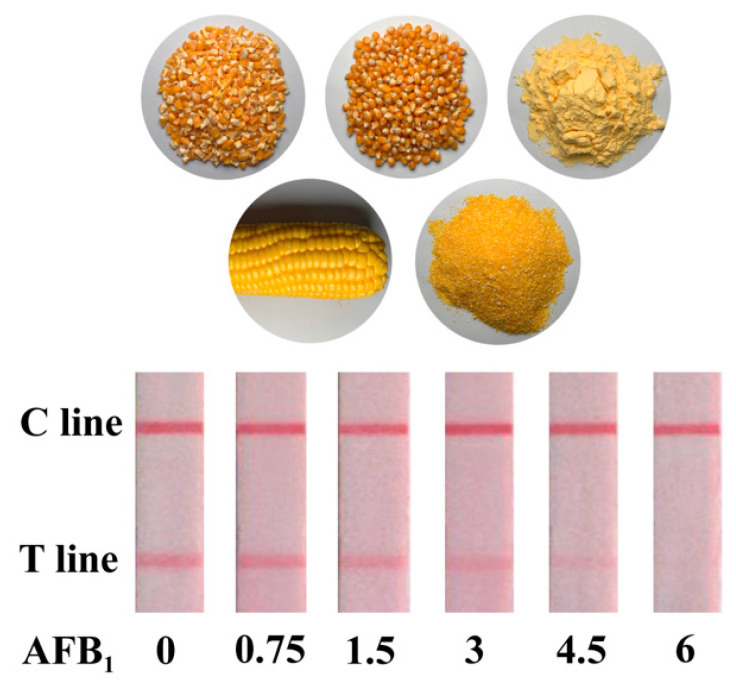
Detection result of AFB_1_ in maize samples. vLOD and cut-off value were present by the PMs-ICA, respectively.

The sensitivity and specificity are important factors to evaluate the performance of ICA. Hence, under the optimal condition, the sensitivity and dynamic interval of PMs-ICA in the buffer were determined by testing a series of concentrations, AFB_1_ standards, from 0 to 1 ng/mL. The color intensity of the T line of PMs-ICA decreased until it disappeared with the concentration of the AFB_1_ increase. Generally, the cut-off value was defined as the concentration of AFB_1_ resulting in no red bands on the T line, judged by the naked eye. The results (Figure 3) show that the cut-off value was 1 ng/mL. Furthermore, the color intensity of the T line was calculated with the software TotalLab TL120, and the B/B_0_ value was calculated. The calibration curve fitted by OriginPro 9.1 software (OriginLab Corp., Northampton, MA, USA) was obtained by plotting B/B_0_ against the concentration of AFB_1_. The qLOD was defined as the concentration giving 80% B/B_0_ from the calibration curve. As shown in Figure 3 the calibration curve presented an inverse linear relationship with a high correlation coefficient (R^2^ = 0.9957), and the qLOD was 0.27 ng/mL, derived from the calibration curve. In addition, as shown in Table 1, the PMs-ICA for detection AFB_1_ has better sensitivity and simpler operation than other LFICAs, based on nanoparticles such as colloidal gold nanoparticles, fluorescent microspheres, quantum dots, etc. [37,38,39,40], and the results could be obtained by the naked eye compared to fluorescence sensors. For the sensitivity of PMs-ICA in real samples, the maize samples (Figure 4) were tested. The blank maize samples confirmed by LC–MS/MS were prepared according to the sample preparation listed in Section 2.5. Briefly, 5 g maize samples were extracted with 10 mL of methanol/water (7:3, *v/v*) and centrifuged; the supernatant was diluted three times by PB to eliminate matrix effects. The sensitivity was also determined after spiking with AFB_1_ at concentrations of 0, 0.75, 1.5, 3, 4.5, 6 µg/kg in maize samples using the same method as in PB. As shown in Figure 4 and Figure S2, the cut-off value was 6 µg/kg, and the qLOD was 1.43 µg/kg.

To evaluate the specificity of the PMs-ICA, AFB_2_, AFG_1_, AFM_1_, ZEN, OTA, DON, FB_1_ and T-2 toxins, which were often found in maize, were chosen as interfering compounds. The results were shown in Table 2, and these results indicated the PMs-ICA for AFB_1_ detection would not be affected by other mycotoxins.

### 3.4. Accuracy and Precision

For the accuracy and precision, blank maize samples were spiked with 1, 3, 5, 12 µg/kg in maize samples (Table 3). Recovery of PMs-ICA for detection of AFB_1_ in maize samples was from 96.0% to 107.6% with corresponding CV of 2.5% to 8.4%. These results indicate that PMs-ICA has good accuracy and reproducibility for the detection of AFB_1_ in maize samples.

### 3.5. Detection of Real Maize Samples

To further evaluate the suitability of PMs-ICA for screening in maize samples, 20 real maize samples were detected by PMs-ICA and LC–MS/MS. Among these samples, four samples (nos. 6, 9, 14, and 18) detected by PMS were 1.1, 1.2, 0.7, 1.9, respectively, whereas those detected by LC–MS/MS were 1.2, 1.1, 0.9, 1.8, respectively. Other samples were not detected by either method (Table 4). The detection demonstrated that the developed PMs-ICA would be reliable for detection of AFB_1_ in maize samples.

## 4. Conclusions

In summary, an ICA, based on PMs, was developed for sensitive and quantitative detection of AFB_1_ in maize. The PMs-ICA was more sensitive than CG-ICA and could obtain results by the naked eye instead of utilizing a reader, such as the FMs-ICA. The developed PMs-ICA showed the cut-off value of 1 ng/mL in PB and 6 µg/kg in maize samples, respectively. The qLOD was 0.27 and 1.43 µg/kg in PB and maize samples, respectively. The accuracy and precision of the PMs-ICA were evaluated by analysis of spiked maize samples with recoveries of 96.0% to 107.6% and coefficients of variation below 10%. In addition, the reliability of PMs-ICA was confirmed by the LC–MS/MS method. The results indicated that the PMs-ICA could be used as a sensitive, simple, rapid point-of-care testing of AFB_1_ in maize.

## Figures and Tables

**Table 1:** Comparison of performances and convenience of LFICAs for AFB_1_ detection.

Nanoparticles	Cut-Off Value (ng/mL)	Result Judging	References
Colloidal gold	10	Naked eye	[37]
Quantum dot	1	Reader	[38]
Fluorescent microspheres	2.5	Reader	[39]
Time-resolved fluorescent Nanobeads	>1	Reader	[40]
PMs	1	Naked eye	this work

**Table 2:** The specificity of PMs-ICA for detection of AFB_1_.

Mycotoxin	IC_50_	CR (%)
AFB_1_	0.57	100
AFB_2_	2.56	22.3
AFG_1_	3.90	14.6
AFG_2_	15.83	3.6
AFM_1_	1.34	42.5
OTA	>10,000	<0.1
DON	>10,000	<0.1
FB_1_	>10,000	<0.1
T-2	>10,000	<0.1

**Table 3:** Accuracy and precision of PMs-ICA for detection of AFB_1_ in maize samples (*n* = 3).

Samples	Added (µg/kg)	Found (µg/kg)	Recovery (%)	CV (%)
Maize	1	1.05	105.0	8.4
	3	2.88	96.0	3.2
	5	5.38	107.6	2.5
	12	+ ^1^	/ ^2^	/

^1^ positive; ^2^ unable to calculate.

**Table 4:** Detection result of real maize samples using PMs-ICA and LC–MS/MS (*n* = 3).

Sample Number	PMs-ICA (µg/kg)	LC–MS/MS
1	ND ^1^	ND
2	ND	ND
3	ND	ND
4	ND	ND
5	ND	ND
6	1.1	1.2
7	ND	ND
8	ND	ND
9	1.2	1.1
10	ND	ND
11	ND	ND
12	ND	ND
13	ND	ND
14	0.7	0.9
15	ND	ND
16	ND	ND
17	1.9	1.8
18	ND	ND
19	ND	ND
20	ND	ND

^1^ ND: Not detected.

## Data Availability

Not applicable.

## Supplementary Materials

The following are available online at https://www.mdpi.com/article/10.3390/bios11060200/s1, Figure S1: Optimization of other parameters in the PMs-ICA. (A) The time of PMs couple with mAb (15, 30, 45, 60 min). (B) The time of blocking (15, 30, 45, 60 min), Figure S2: Analytical sensitivity of PMs-ICA for the detection of AFB_1_ in maize, Table S1: Optimal conditions for PMs couple with mAb.

## References

[1] Beizaei A., O’Kane S.L., Kamkar A., Misaghi A., Henehan G., Cahill D.J. (2015). Highly sensitive toxin microarray assay for aflatoxin B1 detection in cereals. Food Control.

[2] Chun H.S., Kim H.J., Ok H.E., Hwang J.-B., Chung D.-H. (2007). Determination of aflatoxin levels in nuts and their products consumed in South Korea. Food Chem..

[3] Rawal S., Kim J.E., Coulombe R. (2010). Aflatoxin B1 in poultry: Toxicology, metabolism and prevention. Res. Vet. Sci..

[4] Sun L., Zhao Q. (2018). Direct fluorescence anisotropy approach for aflatoxin B1 detection and affinity binding study by using single tetramethylrhodamine labeled aptamer. Talanta.

[5] Wu F., Groopman J.D., Pestka J.J. (2014). Public health impacts of foodborne mycotoxins. Annu. Rev. Food Sci. Technol..

[6] Bedard L.L., Massey T.E. (2006). Aflatoxin B1-induced DNA damage and its repair. Cancer Lett..

[7] Li Z., Xu X., Fu Y., Guo Y., Zhang Q., Zhang Q., Yang H., Li Y. (2019). A water-stable luminescent metal–organic framework for effective detection of aflatoxin B1 in walnut and almond beverages. RSC Adv..

[8] European Commission (2006). Commission Regulation (EC) No 1881/2006 of 19 December 2006 setting maximum levels for certain contaminants in foodstuffs. Off. J. Eur. Union.

[9] Khodaei D., Javanmardi F., Khaneghah A.M. (2021). The global overview of the occurrence of mycotoxins in Cereals: A three-year survey. Curr. Opin. Food Sci..

[10] Jha S.N., Jaiswal P., Kaur J., Ramya H. (2021). Rapid Detection and Quantification of Aflatoxin B1 in Milk Using Fourier Transform Infrared Spectroscopy. J. Inst. Eng. (India) Ser. A.

[11] Sakin F., Tekeli İ.O., Yipel M., Kürekci C. (2018). Occurrence and health risk assessment of aflatoxins and ochratoxin a in Sürk, a Turkish dairy food, as studied by HPLC. Food Control.

[12] Jangampalli Adi P., Matcha B. (2018). Analysis of aflatoxin B1 in contaminated feed, media, and serum samples of Cyprinus carpio L. by high-performance liquid chromatography. Food Quality Saf..

[13] Du X., Schrunk D.E., Imerman P.M., Smith L., Francis K., Tahara J., Tkachenko A., Reimschuessel R., Rumbeiha W.K. (2019). Evaluation of a diagnostic method to quantify aflatoxins B1 and M1 in animal liver by high-performance liquid chromatography with fluorescence detection. J. AOAC Int..

[14] Nakhjavan B., Ahmed N.S., Khosravifard M. (2020). Development of an improved method of sample extraction and quantitation of multi-mycotoxin in feed by LC-MS/MS. Toxins.

[15] Dada T.A., Ekwomadu T.I., Mwanza M. (2020). Multi mycotoxin determination in dried beef using liquid chromatography coupled with triple quadrupole mass spectrometry (LC-MS/MS). Toxins.

[16] Mahmoudi T., de la Guardia M., Baradaran B. (2020). Lateral flow assays towards point-of-care cancer detection: A review of current progress and future trends. TrAC Trends Anal. Chem..

[17] Hnasko R.M., Jackson E.S., Lin A.V., Haff R.P., McGarvey J.A. (2021). A Rapid and Sensitive Lateral Flow Immunoassay (LFIA) for the Detection of Gluten in Foods. Food Chem..

[18] Guo X., Yuan Y., Liu J., Fu S., Zhang J., Mei Q., Zhang Y. (2021). Single-Line Flow Assay Platform Based on Orthogonal Emissive Upconversion Nanoparticles. Anal. Chem..

[19] Zhang Y., Xu J., Zhou S., Zhu L., Lv X., Zhang J., Zhang L., Zhu P., Yu J. (2020). DNAzyme-triggered visual and ratiometric electrochemiluminescence dual-readout assay for Pb (II) based on an assembled paper device. Anal. Chem..

[20] Kim H., Chung D.-R., Kang M. (2019). A new point-of-care test for the diagnosis of infectious diseases based on multiplex lateral flow immunoassays. Analyst.

[21] Wang X., Xiong E., Tian T., Cheng M., Lin W., Wang H., Zhang G., Sun J., Zhou X. (2020). Clustered regularly interspaced short palindromic repeats/Cas9-mediated lateral flow nucleic acid assay. ACS Nano.

[22] Zhu C., Zhang G., Huang Y., Yang S., Ren S., Gao Z., Chen A. (2018). Dual-competitive lateral flow aptasensor for detection of aflatoxin B1 in food and feedstuffs. J. Hazard. Mater..

[23] Cheng N., Song Y., Shi Q., Du D., Liu D., Luo Y., Xu W., Lin Y. (2019). Au@ Pd nanopopcorn and aptamer nanoflower assisted lateral flow strip for thermal detection of exosomes. Anal. Chem..

[24] Razo S.C., Panferov V.G., Safenkova I.V., Varitsev Y.A., Zherdev A.V., Dzantiev B.B. (2018). Double-enhanced lateral flow immunoassay for potato virus X based on a combination of magnetic and gold nanoparticles. Anal. Chim. Acta.

[25] Dzantiev B.B., Byzova N.A., Urusov A.E., Zherdev A.V. (2014). Immunochromatographic methods in food analysis. TrAC Trends Anal. Chem..

[26] Xie Q.-Y., Wu Y.-H., Xiong Q.-R., Xu H.-Y., Xiong Y.-H., Liu K., Jin Y., Lai W.-H. (2014). Advantages of fluorescent microspheres compared with colloidal gold as a label in immunochromatographic lateral flow assays. Biosens. Bioelectron..

[27] Li X., Chen X., Liu Z., Wang J., Hua Q., Liang J., Shen X., Xu Z., Lei H., Sun Y. (2021). Latex microsphere immunochromatography for quantitative detection of dexamethasone in milk and pork. Food Chem..

[28] Borse V., Srivastava R. (2019). Fluorescence lateral flow immunoassay based point-of-care nanodiagnostics for orthopedic implant-associated infection. Sens. Actuators B Chem..

[29] Li S.-J., Sheng W., Wen W., Gu Y., Wang J.-P., Wang S. (2018). Three kinds of lateral flow immunochromatographic assays based on the use of nanoparticle labels for fluorometric determination of zearalenone. Microchim. Acta.

[30] Taranova N., Berlina A., Zherdev A., Dzantiev B. (2015). ‘Traffic light’immunochromatographic test based on multicolor quantum dots for the simultaneous detection of several antibiotics in milk. Biosens. Bioelectron..

[31] Panferov V.G., Safenkova I.V., Zherdev A.V., Dzantiev B.B. (2020). Urchin peroxidase-mimicking Au@ Pt nanoparticles as a label in lateral flow immunoassay: Impact of nanoparticle composition on detection limit of Clavibacter michiganensis. Microchim. Acta.

[32] Zhang H., Wang L., Yao X., Wang Z., Dou L., Su L., Zhao M., Sun J., Zhang D., Wang J. (2020). Developing a Simple Immunochromatography Assay for Clenbuterol with Sensitivity by One-Step Staining. J. Agric. Food Chem..

[33] Zhang X., Yu X., Wen K., Li C., Mujtaba Mari G., Jiang H., Shi W., Shen J., Wang Z. (2017). Multiplex lateral flow immunoassays based on amorphous carbon nanoparticles for detecting three fusarium mycotoxins in maize. J. Agric. Food Chem..

[34] Song S., Liu N., Zhao Z., Njumbe Ediage E., Wu S., Sun C., De Saeger S., Wu A. (2014). Multiplex lateral flow immunoassay for mycotoxin determination. Anal. Chem..

[35] Liu Z., Hua Q., Wang J., Liang Z., Li J., Wu J., Shen X., Lei H., Li X. (2020). A smartphone-based dual detection mode device integrated with two lateral flow immunoassays for multiplex mycotoxins in cereals. Biosens. Bioelectron..

[36] Li X., Wang J., Yi C., Jiang L., Wu J., Chen X., Shen X., Sun Y., Lei H. (2019). A smartphone-based quantitative detection device integrated with latex microsphere immunochromatography for on-site detection of zearalenone in cereals and feed. Sens. Actuators B Chem..

[37] Chen Y., Chen Q., Han M., Zhou J., Gong L., Niu Y., Zhang Y., He L., Zhang L. (2016). Development and optimization of a multiplex lateral flow immunoassay for the simultaneous determination of three mycotoxins in corn, rice and peanut. Food Chem..

[38] Jia B., Liao X., Sun C., Fang L., Zhou L., Kong W. (2021). Development of a quantum dot nanobead-based fluorescent strip immunosensor for on-site detection of aflatoxin B1 in lotus seeds. Food Chem..

[39] Liu D., Huang Y., Chen M., Wang S., Liu K., Lai W. (2015). Rapid detection method for aflatoxin B1 in soybean sauce based on fluorescent microspheres probe. Food Control.

[40] Wang X., Wu X., Lu Z., Tao X. (2020). Comparative Study of Time-Resolved Fluorescent Nanobeads, Quantum Dot Nanobeads and Quantum Dots as Labels in Fluorescence Immunochromatography for Detection of Aflatoxin B1 in Grains. Biomolecules.

