# Supplementary material for: Polystyrene Microsphere-Based Immunochromatographic Assay for Detection of Aflatoxin B1 in Maize
Source: Biosensors (Basel). 2021 Jun 20;11(6):200. doi: 10.3390/bios11060200 (PMC8234612; doi:10.3390/bios11060200)
Supplement: Supplementary file 1 [file biosensors-11-00200-s001.zip › biosensors-1223578-supplementary.pdf]

Polystyrene microspheres based immunochromatographic assay for detection of Aflatoxin B_1 in maize

Jin Wang [1,†], Xiangmei Li [1,†], Xing Shen [1], Ang Zhang [2], Jinxiu Liu [1] and Hongtao Lei [1,3,*]

[1]Guangdong Provincial Key Laboratory of Food Quality and Safety, South China Agricultural University, Guangzhou 510642, China; wangjin940810@stu.scau.edu.cn (J.W.); lixiangmei12@163.com (X.L.); shenxing325@163.com (X.S.); ljx199108z@163.com (J.L.)

[2]Technology Center of Qinhuangdao Customs, Qinhuangdao 066004, China; zhanganggrape@hotmail.com

[3]Guangdong Laboratory for Lingnan Modern Agriculture, Guangzhou 510642, China

[*]Correspondence: hongtao@scau.edu.cn; Tel.: +86-20-8528-3925

[†]Jin Wang and Xiangmei Li have equally contributed to this work.

Preparation of coating antigen and anti-AFB_1 mAb

Preparation of coating antigen and immunogen

1 mg AFB_1 was dissolved in 0.6 mL methanol/water/pyridine solution (4:1:1, v/v), 2 mg carboxymethoxylamine hemihydrochloride (CMO) was added, and the reaction was stirred at 70°C water bath. After the reaction, the mixture was blown dry with nitrogen, the precipitate was dissolved in 1 mL of $CHCl_3$ solution, and the same volume of ultrapure water was extracted three times, the organic phase was collected and dry with nitrogen. The precipitate is dissolved in 200 μL DMF solution to obtain the hapten of AFB_1-CMO solution.

The above AFB_1-CMO solution was added 2.5 mg of EDC and 1.5 mg NHS, and the mixture reacted with stirring at room temperature for 12 h. The keyhole limpet haemocyanin (KLH) solution was added dropwise to the above solution and reacted overnight at room temperature. The reaction mixture was dialyzed against PBS (pH 7.4, 10 mM) at 4°C for 3 days, the immunogen AFB1-CMO-KLH was obtained.

The coating antigen AFB1-CMO-BSA was obtained by the method described above, but KLH was replaced by BSA.

Preparation of anti-AFB_1 mAb

The process of animal immunization and cell fusion was described in our previous publication [1]. The most sensitive hybridoma was cultured to produce ascites. The protein G resin was used to yield the mAb, which was stored at -20°C.

Optimization of other parameters of PMs-ICA

The time of PMs couple mAb determines the amount of mAb coupled on PMs to affect the sensitivity of PMs-ICA. 15, 30, 45, 60 min were considered and tested by the negative control groups (PBS, 0.01mol/L, pH 7.4) and positive test groups (1 ng/mL AFB_1). As shown in Figure S1A, for the negative control groups, the color intensity of T lines at 30 min was stronger than at 15 min, and was almost as same as the color intensity of T line at strong at 45 min and 60 min. For the positive test groups, the color intensity of T lines was the same and colorless. Considering the sensitivity and time, coupling 30 min was selected.

The time of blocking PMs to bind the site to the mAb affected non-specific adsorption. In order to reduce non-specific adsorption and the time of blocking. 15, 30, 45, 60 min were selected to test by the negative control groups (PBS, 0.01mol/L, pH 7.4) and positive test groups (1 ng/mL AFB_1). As shown in Figure S1B, the color

intensity of T lines and inhibition results were the same in PMs-ICA at 15, 30, 45, 60 min by testing of negative control groups and positive test groups. Therefore, 15 min for blocking was selected it took less time.

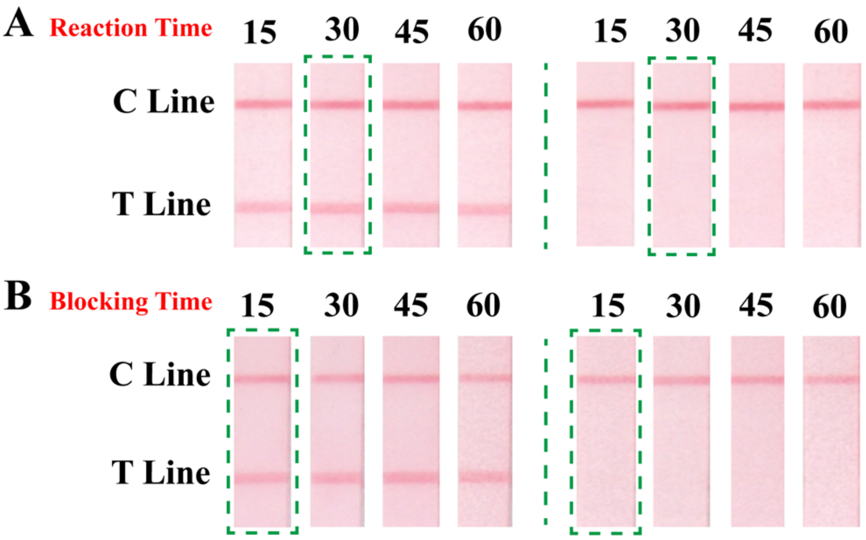

Figure S1. Optimization of other parameters in the PMs-ICA. (A) The time of PMs couple with mAb (15, 30, 45, 60 min). (B) The time of blocking (15, 30, 45, 60 min).

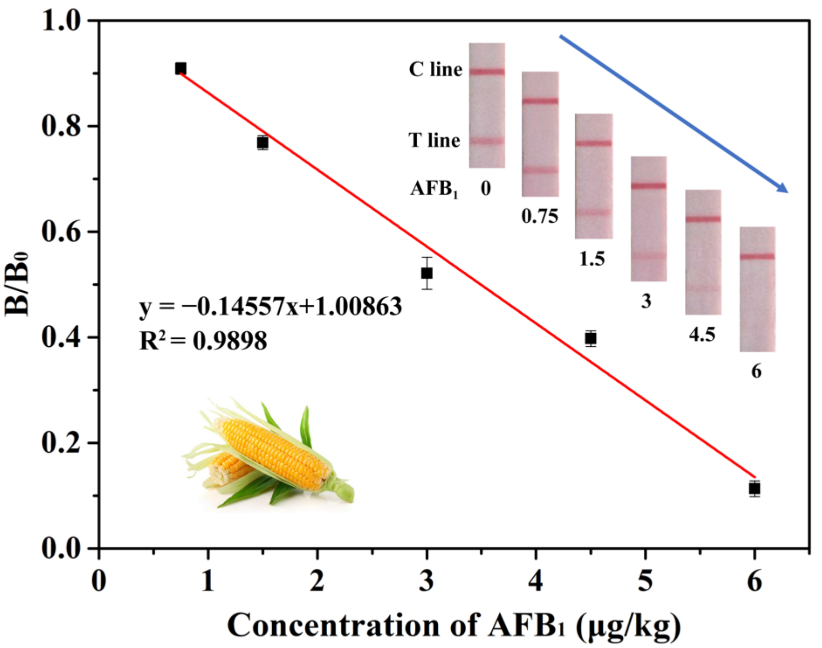

Figure S2. Analytical sensitivity of PMs-ICA for the detection of AFB_1 in maize.

Table S1. Optimal conditions for PMs couple with mAb

Item	Optimal condition
The mount of PMs	15 μL
The condition for activating carboxyl groups	MES (0.05 mol/L, pH 5.5)
Coupling time	30 min
The mount of mAb	3 μL
Blocking time	15 min
The volume of immune probe	4 μL

Reference

1. Chen, Z.-J.; Liu, X.-X.; Xiao, Z.-L.; Fu, H.-J.; Huang, Y.-P.; Huang, S.-Y.; Shen, Y.-D.; He, F.; Yang, X.-X.; Hammock, B. Production of a specific monoclonal antibody for 1-naphthol based on novel hapten strategy and development of an easy-to-use ELISA in urine samples. *Ecotoxicology and environmental safety* **2020**, *196*, 110533.
